# Prognostic Implications and Immune Infiltration Characteristics of Chromosomal Instability-Related Dysregulated CeRNA in Lung Adenocarcinoma

**DOI:** 10.3389/fmolb.2022.843640

**Published:** 2022-03-28

**Authors:** Shengnan Guo, Tianhao Li, Dahua Xu, Jiankai Xu, Hong Wang, Jian Li, Xiaoman Bi, Meng Cao, Zhizhou Xu, Qianfeng Xia, Ying Cui, Kongning Li

**Affiliations:** ^1^ Key Laboratory of Tropical Translational Medicine of Ministry of Education, College of Biomedical Information and Engineering, Institute of Nephrology Second Affiliated Hospital and Hainan General Hospital, Hainan Medical University, Haikou, China; ^2^ College of Bioinformatics Science and Technology, Cancer Hospital, Harbin Medical University, Harbin, China; ^3^ Key Laboratory of Tropical Translational Medicine of Ministry of Education, NHC Key Laboratory of Control of Tropical Diseases, School of Tropical Medicine, The Second Affiliated Hospital, Hainan Medical University, Haikou, China

**Keywords:** chromosomal instability, dysregulated ceRNA, immune microenvironment, prognosis, lung adenocarcinoma

## Abstract

An accumulating body of research indicates that long-noncoding RNAs (lncRNAs) regulate the target genes and act as competitive endogenous RNAs (ceRNAs) playing an indispensable role in lung adenocarcinoma (LUAD). LUAD is frequently accompanied by the feature of chromosomal instability (CIN); however, CIN-related ceRNAs have not been investigated yet. We systematically analyzed and integrated CIN-related dysregulated ceRNAs characteristics in LUAD samples for the first time. In TCGA LUAD cohort, CIN in tumor samples was significantly higher than that in those of adjacent, and patients with high CIN risk tended to have worse clinical outcomes. We constructed a double-weighted CIN-related dysregulated ceRNA network, in which edge weight and node weight represented the disorder extent of ceRNA and the correlation of RNA expression level and prognosis, respectively. After module mining and analysis, a potential prognostic biomarker composed of 12 RNAs (8 mRNAs and 4 lncRNAs) named CIN-related dysregulated ceRNAs (CRDC) was obtained. The CRDC risk score had a positive relation with clinical stage and CIN, and patients with high CRDC risk scores exhibited poor prognosis. Moreover, CRDC tended to be an independent risk factor with high robustness to overcome the effect of multicollinearity among other explanatory variables for disease-specific survival (DSS) in TCGA and two GEO cohorts. The result of functional analysis indicated that CRDC was involved in multiple cancer progresses, especially immune-related pathways. The patients with lower CRDC risk had higher B cell, T cell CD4^+^, T cell CD8^+^, neutrophil, macrophage, and myeloid dendritic cell infiltration than the patients with higher CRDC risk. Meanwhile, patients with lower CRDC risk could get more benefits from immunological therapy. The results suggested that the CRDC could be a potential prognostic biomarker and an immunotherapy predictor for lung adenocarcinoma.

## Introduction

Lung cancer is one of the leading causes of cancer-related deaths in both men and women worldwide, with LUAD, a kind of non-small-cell lung cancer (NSCLC), accounting for a large proportion (Reck and Rabe, 2017). CIN, the major type of genomic instability including gain/loss of whole chromosomes or large segments (aneuploidy), structural rearrangements, and focal aberrations (Geigl et al., 2008), confers tumorigenesis, metastasis, drug resistance, and poor prognosis (Lee et al., 2011; Duijf and Benezra, 2013; Bakhoum and Cantley, 2018; Bakhoum et al., 2018). CIN can be effectively detected and tended to be a risk factor for poor prognosis in LUAD (Choi et al., 2009), which mediates intratumor heterogeneity, therefore increasing the risk of recurrence or death (Jamal-Hanjani et al., 2017). CIN may counteract the therapeutic effectiveness of oncogene withdrawal treatment and be responsible for tumor relapse in lung cancer (Sotillo et al., 2010).

LncRNAs indirectly regulate target mRNAs by sharing common microRNA response elements (MREs) formatting a posttranscriptional mechanism: competing endogenous RNA (ceRNA) (Salmena et al., 2011). CeRNAs play an indispensable role in the development of carcinogenesis and could be detected based on multiple computational methods (Le et al., 2017; Zhang et al., 2019) An oncogenic lncRNA HOTAIR and a protein-coding gene HER2 associated with gastric carcinogenesis regulated each other owing to inhibition from miR-331-3p, which provides a potential anticancer treatment scheme (Liu et al., 2014). LncRNA WDFY3-AS2 and mRNA RORA are both involved in suppression of ovarian cancer by the WDFY3-AS2/miR-18a/RORA axis, in which miR-18a is reported to be an oncogene (Li W. et al., 2020). Given the ceRNA crosstalk exhibits reciprocal and complexity features, researchers focused on the complicated ceRNA regulation network to exploit cancer-associated key molecules (Sumazin et al., 2011; Karreth and Pandolfi, 2013; Song et al., 2017). Perturbation of ceRNA interaction wildly exists in disease versus normal status. LncRNAs with ceRNA activity could be candidate epigenetic diagnostic biomarkers for early detection of osteoporosis by constructing an osteoporosis-related dysregulated ceRNA network (Zhang et al., 2020). Also, epigenetically related lncRNAs involved in dysregulated ceRNA–ceRNA networks offered novel potential molecular therapeutic targets across pan-cancer (Xu et al., 2021). However, the molecular function of dysregulated ceRNA in LUAD remains further elucidated.

Based on the molecular pattern, immunotherapy has become a new treatment option for NSCLC in recent years (Reck and Rabe, 2017). The blockade of immune checkpoints targeting cytotoxic T lymphocyte—associated antigen 4 (CTLA4) and the programmed cell death protein 1 pathway (PD-1/PD-L1) have demonstrated promise in stimulating antitumor immunity (Pardoll, 2012). Existing research studies have shown that clinical outcome and treatment response to immune checkpoint blockers are affected by the composition and proportion of various immune cells (Brahmer, 2013; Schalper et al., 2015; Kadara et al., 2017). A vital element in precision diagnosis and personalized treatment of LUAD is exploring novel molecular signatures, especially those associated with the tumor immune microenvironment. Moreover, the ceRNA regulation mechanism could affect immune cell infiltration in multiple cancers. For instance, LINC00301 could serve as a competing endogenous RNA (ceRNA) against miR-1276 to expedite the HIF1α pathway in the cytoplasm of NSCLC cells facilitating tumor progression and triggering an immune-suppressing microenvironment (Sun et al., 2020). SNHG16 serves as a ceRNA by sponging miR-16-5p, which led to the derepression of its target gene SMAD5 and resulted in potentiation of the TGF-β1/SMAD5 pathway to induce immunosuppressive CD73+γδ1 Treg cells (Ni et al., 2020). So far, no studies have systematically excavated the role of CIN-related ceRNAs in immune regulation for LUAD patients.

**FIGURE 1:**
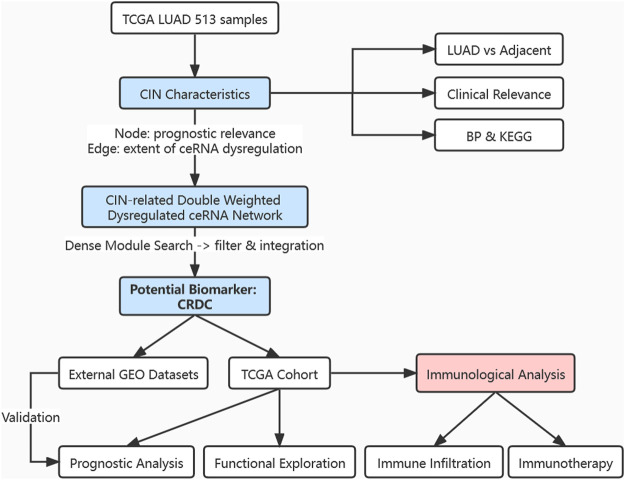
Flowchart of this research. Exploration of CIN feature in expression level, clinical outcome relevance, and functional analysis; construction of a CIN-related double-weighted dysregulated ceRNA network; acquisition of a potential biomarker CRDC after dense module searching, filtering, and integration; analyzation and evaluation of CRDC in the prognostic value; inference for immune cell infiltration and benefit from immunotherapy.

Here, we analyzed the CIN features in LUAD samples and systematically integrated CIN-related ceRNA characteristics for the first time by constructing a double-weighted CIN-related dysregulated ceRNA network, aiming to identify a potential biomarker for LUAD prognosis. The marker we found was assessed combined with multiple clinical factors. Furthermore, it was associated with immune infiltration which may help predict whether patients will benefit from immunotherapy (Figure 1). This research provided a perspective for identifying prognostic markers and exploring the immune microenvironment characteristics of LUAD patients.

## Materials and Methods

### Data Acquisition and Preprocessing

LUAD HTSeq transcriptome profiles were acquired from TCGA data portal (http://cancergenome.nih.gov/, Data Release 9.0) (Tomczak et al., 2015). The GENCODE V22 (GRCh38) (Frankish et al., 2019) human genome annotation file was used to annotate mRNA and lncRNA transcripts. The gene types of “processed_transcript,” “lincRNA,” “3prime_overlapping_ncrna,” “antisense,” “non_coding,” “sense_intronic,” and “sense_overlapping” with length greater than 200 nt were defined as lncRNAs. The transcripts per kilobase million (TPM) value was converted from fragments per kilobase million (FPKM) according to the formula: $TPM=\frac{FPKM}{\sum_{all\;genes}FPKM}*10^{6}$ (Li and Dewey, 2011). TPM transcriptome profiles were further normalized by log2(x+1) transformed, and the genes covering >30% missing values were filtered. We adopted clinic information from the work of Liu et al., which provided the usage recommendations for corresponding TCGA LUAD cohort in the meanwhile (Liu et al., 2018). Gene expression profiles and clinical information from two GEO datasets were collected as the external validation datasets (Supplementary Table S1).

### MiRNA–Target Regulation Relationships

Experimentally validated miRNA–mRNA interaction information was collected from miRTarBase 7.0 (Chou et al., 2018), miRecords 2013 (Xiao et al., 2009), and TarBase 6.0 (Vergoulis et al., 2012). A total of 388,895 unique interactions that contained 2,846 miRNAs and 18,936 mRNAs were obtained. We integrated starBase 2.0 (Li J.-H. et al., 2014), DIANA-LncBase V2 (Paraskevopoulou et al., 2016), and lncRNASNP2 (Miao et al., 2018) databases and obtained 10,318 experimentally validated nonredundant miRNA–lncRNA interactions involving 290 miRNAs and 1,162 lncRNAs.

### Estimation of the Chromosomal Instability Risk Score of Samples

Chromosomal instability (CIN) risk scores of samples were calculated by summing of the CIN70 genes’ TPM value, whose expression level was consistently correlated with total functional aneuploidy in several cancer types (Carter et al., 2006). A high CIN score indicates a high extent of chromosomal instability.

### Construction of a Chromosomal Instability-Related Dysregulated CeRNA Network

1) CeRNA Identification in LUAD CIN-Low and CIN-High Patients

Differentially expressed genes between tumor and normal adjacent samples were reserved for identifying ceRNA pairs in LUAD CIN-Low and CIN-High patients (Zhou et al., 2014; Xu et al., 2015). First, we screened mRNA–lncRNA pairs which shared significantly common upstream miRNA regulation according to the hypergeometric test FDR-adjusted *p* value <0.05. The *p* value was calculated as $1-\overset{r-1}{\underset{i=0}{\sum }}\frac{\left(\begin{array}{c} K\\ i \end{array}\right)\left(\begin{array}{c} N-K\\ M-i \end{array}\right)}{\left(\begin{array}{c} N\\ M \end{array}\right)}$ , where *N* was the number of background human miRNAs, *M* and *K* represented the number of miRNAs regulating the current mRNA and lncRNA, respectively, and *r* was the common miRNA number between the two RNAs. Also, Pearson correlation coefficients (PCC) > 0.1 as well as correlation FDR-adjusted *p* value <0.05 were considered significantly co-expressed pairs for having a similar expression trend. For CIN-Low and CIN-High LUAD patients’ groups separately, mRNA–lncRNA pairs that met the aforementioned conditions of co-regulated and co-expressed RNAs were recognized as ceRNAs which served as nodes in the dysregulated network.

2) CIN-Related Dysregulated CeRNA Network

In the dysregulated ceRNA network, alteration of ceRNA PCC between the CIN-High group and CIN-Low group in the LUAD cohort was assigned to measure the weight of edges and the node weight quantifying the prognostic role of the gene for LUAD DSS (Li et al., 2012; Wang et al., 2015). Given a ceRNA pair, $r_{CIN\_High}$ and $r_{CIN\_Low}$ represented the PCC in two groups, and $n_{CIN\_High}$ and $n_{CIN\_Low}$ represented the sample size. If the ceRNA identification conditions were not satisfied in one of the CIN-Low and CIN-High groups, the PCC was set to 0. The edge weight (E) was calculated as $\varphi^{-1}\left[1-2*\left(1-\varphi \left(\left|\frac{F\left(r_{CIN\_High}\right)-F\left(r_{CIN\_Low}\right)}{\sqrt{\frac{1}{n_{CIN\_High}+3}+\frac{1}{n_{CIN\_Low}+3}}}\right|\right)\right)\right]$ , where $F\left(x\right)=\frac{1}{2}ln\frac{1+x}{1-x}$ , and φ denoted the standard normal distribution function. Meanwhile, the node weight (N) of the network was defined as $\varphi^{-1}\left(1-p\right)$ , where *p* was the univariate Cox proportional hazards analysis significant *p* value, adopting the samples without postoperative treatment for purpose of eliminating its effect on clinical outcome.

### Dense Modules Identification and Filtration

1) Searching for Dense Modules in Dysregulated CeRNA Network

The objective of building a dysregulated network was to identify dense modules with relatively high weight of node and edge. We utilized the algorithm from the dmGWAS_3.0 R package (Wang et al., 2015) to define the module score and conduct module mining. The module score was $\lambda \frac{\sum E}{\sqrt{n_{E}}}+\left(1-\lambda \right)\frac{\sum N}{\sqrt{n_{N}}}$ , where $n_{E}$ and $n_{N}$ were the number of edges and nodes in the network, and λ was between 0 and 1. The method of module search are summarized into the following three parts: 1) For each gene in the network, it was assigned as a seed module at the beginning; 2) add a neighbor node whose shortest path to any node in the module was shorter or equal to *d* into the module once at a time only when the increment of the current module score was greater than *r* multiply by the score of the previous module; 3) repeat these steps till no more neighbors could be added. The parameter *d* was decided as 1, *r* = 0.1, and *λ* was set to 0.44.

2) Screening and Integrating Dense Modules

In order to seek valuable messages from a large number of dense modules, we prioritized network modules by following three qualification rules. First, the top 10% modules with the highest scores were selected for further analysis. Second, a random perturbation strategy was used to screen out the non-randomized prognostic modules by multivariate Cox regression analysis for DSS. For each given module, random multivariate Cox regression *p* values were calculated after random acquisition of the same number of genes in the background 10,000 times. Then, sorting the 10,000 *p* values in ascending order and only the modules ranked in top 1% (random *p* value <0.01) were left. Furthermore, we merged the modules via genes in common and acquired the subnetwork named CIN-related dysregulated ceRNAs (CRDC).

### Calculation of the CIN-Related Dysregulated CeRNAs Risk Score

The CRDC risk score of each LUAD patient was constructed by a linear combination of gene expression values and log-transformed hazard ratios (HR) from the univariate Cox regression model, in which the univariate Cox regression analysis was conducted using the DSS without undergoing postoperative treatment. The CRDC risk score was calculated as $\overset{N}{\underset{i=1}{\sum }}Expression_{\_i}\text{*log}\left(HR_{\_i}\right)$ , where N was the number of the CRDC genes.

### Immune Infiltration Analysis in LUAD Patients

The LUAD patients’ immune score, stromal score, and estimate score were calculated by the ESTIMATE R package (version 1.0.13); meanwhile, tumor purity was obtained by cos(0.6049872018 + 0.0001467884 × ESTIMATE score) (Yoshihara et al., 2013). Tumor mutation load (TMB) was performed as non-synonymous mutation counts in coding sequences (CDSs) divided by total number of CDSs then multiplied by 10^6^. The geometric mean of GZMA and PRF1 TPM expression values estimated were defined as cytolytic activity (CYT), which reflected the local immune cytolytic T-cell activity (Rooney et al., 2015). TIMER2.0 (Li T. et al., 2020) was applied to estimate immune cell infiltration levels in the tumor immune microenvironment for LUAD patients. The tumor immune dysfunction and exclusion (TIDE) score, a signature consistent with tumor immune evasion, which could predict immune checkpoint blockade (ICB) response, was performed by TIDE command-line interface with tumor TPM expression normalized by adjacent normal samples (Jiang et al., 2018; Fu et al., 2020). The immunophenoscore (IPS), a superior predictor of response to anti-CTLA4 and anti-PD-1 antibodies, was used to evaluate tumor immunogenicity (Charoentong et al., 2017). Also, we predicted the possible response to immunotherapy by the SubMap method from GenePattern (https://cloud.genepattern.org/gp/pages/index.jsf).

### Statistical Analysis

Differentially expressed genes (DEGs) were identified by the DESeq2 R package (Love et al., 2014) with the threshold of |log2FC| > 1 and FDR <0.01. CIN-High and CIN-Low as well as CRDC-High and CRDC-Low patients were divided by the StepMiner method, which could find an appropriate threshold for the dichotomizing numeric vector (Sahoo et al., 2007; Liu et al., 2013). For example, the CIN scores were sorted in ascending order and were used for finding an optimal location *t* which maximized the signal-to-noise ratio (SNR). $SNR=\frac{\sum_{i=1}^{n}{\left(\mu_{1}I\left(i\leq t\right)+\mu_{2}I\left(i>t\right)-\mu \right)}^{2}}{\sum_{i=1}^{n}{\left(\mu_{1}I\left(i\leq t\right)+\mu_{2}I\left(i>t\right)-CIN_{i}\right)}^{2}},$ where *I* is the indicator function and is set to 0 when the condition in brackets is not satisfied, μ is the mean CIN score for all patients, and $\mu_{1}$ and $\mu_{2}$ represent the mean CIN score of two groups separated by the location *t*. Then, the CIN score of position *t* was regarded as the classification threshold. The conventional ROC curve and time-dependent ROC curve estimation was performed using the pROC (version 1.18.0) (Robin et al., 2011) and survivalROC (version 1.0.3) (Heagerty and Zheng, 2005) R package separately. The Kaplan–Meier survival curves between different groups divided by the CIN score or CRDC score was tested by the log-rank test using the R package survminer (version 0.4.9). The multivariable Cox proportional hazard model, stepwise regression method, and nomogram were constructed by the rms R package (version 6.2–0). The decision curve was built to evaluate benefit of CRDC at different prognostic time points with the DCA R package (version 2.0). Biological progress (BP) enrichment analysis and visualization were implemented by Metascape (Zhou Y. et al., 2019), clusterProfiler R package (version 4.0.5) (Wu et al., 2021), and fgsea R package (version 1.20.0). The hallmark, KEGG pathway, and immunologic gene sets enrichment analysis were performed by the clusterProfiler R package. BP, hallmark, and immunologic gene sets were obtained from MSigDB v7.4 (Subramanian et al., 2005; Liberzon et al., 2015). The R version adopted in this article was 4.0.3. Representation of significance of *p* value in figures were described as follows: *: *p* < 0.05, **: *p* < 0.01, ***: *p* < 0.001, and ns for nonsignificant.

## Results

### Characteristics of Chromosomal Instability in Lung Adenocarcinoma

**FIGURE 2:**
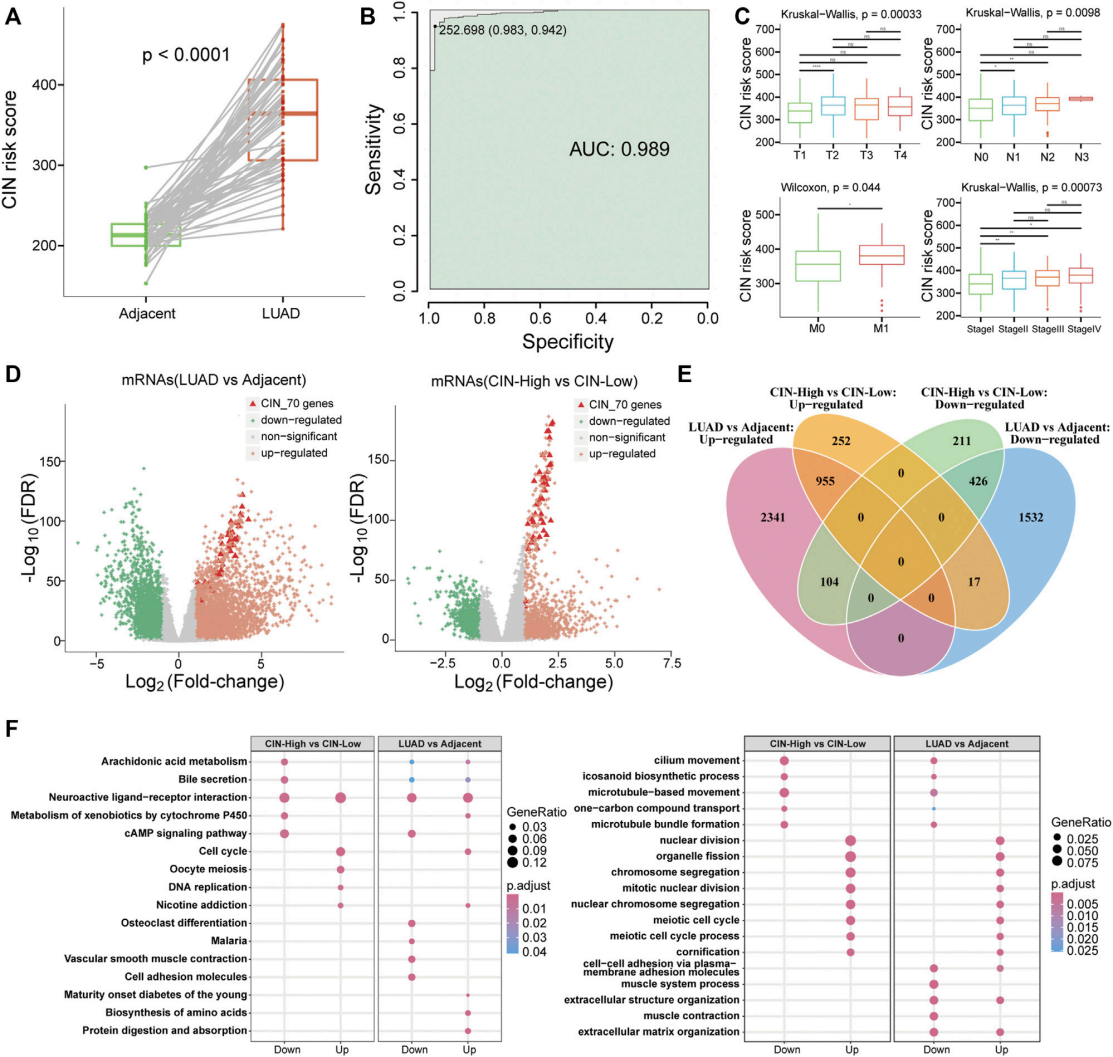
Characteristics of chromosomal instability in lung adenocarcinoma. **(A)** Boxplot for CIN scores in LUAD and adjacent paired samples. The green column indicated adjacent normal samples, and the red column indicated LUAD tissues. **(B)** ROC curve of the CIN score in discriminating LUAD patients from adjacent normal samples. The green color indicated AUC. **(C)** Boxplots of patients’ CIN scores in different TNM stages and clinical stages. **(D)** Volcano plots for identifying significantly differentially expressed genes in (left) lung adenocarcinoma samples versus adjacent samples and (right) CIN-High compared to CIN-Low group. **(E)** Venn diagram and **(F)** BP function and KEGG pathway enrichment results for differentially expressed genes in comparative groups of LUAD vs. Adjacent and CIN-High vs. CIN-Low.

For quantifying chromosomal instability of samples, we calculated CIN scores of all samples by summing up genes’ expression in CIN70 signature. With a paired-sample comparison analysis in 57 LUAD patients, CIN scores of lung adenocarcinoma samples (350.44 ± 60.00) were significantly higher than those of paired adjacent normal samples (214.1201 ± 22.98) (Figure 2A, Wilcoxon signed-rank test, *p* = 5.3e-11) as well as all samples in TCGA LUAD data cohort (Supplementary Figures S1A,B). In 513 LUAD and 59 adjacent normal samples, receiver-operating characteristic (ROC) analysis showed that the CIN score had a high performance in discriminating LUAD patients from adjacent normal samples with the area under the curve (AUC) reached up to 0.989 (Figure 2B). Meanwhile, we found that high CIN scores were related with the worse clinical stage and TNM stage generally (Figure 2C). Also, patients with high CIN scores had worse overall survival (OS), DSS, disease-free interval (DFI), and progression-free interval (PFI) (Supplementary Figures S1C–J). This indicated that LUAD samples possessed higher CIN than adjacent normal samples, which is consistent with the general characteristics of genomic instability in carcinoma (Grady and Carethers, 2008; Martin et al., 2010).

We identified significantly DEGs in LUAD samples by the DESeq2 R package. Within 3,400 upregulated and 1,975 downregulated mRNAs, 52 genes of the CIN70 signature were upregulated, and none of them were downregulated. Similarly, 42 of CIN70 genes showed upregulation in 289 CIN-High patients compared to the CIN-Low group that contained 224 patients. It was observed that these highly altered expressed CIN70 genes in two comparison groups possessed greater extent differential expression than other genes (Figure 2D). Functional enrichment analysis indicated that the DEGs of these two comparable groups shared common biological processes and pathways. For instance, both downregulated gene sets participated in microtubule-based movement and microtubule bundle formation biological progress as well as neuroactive ligand–receptor interaction and cAMP signaling pathways. On the contrary, the upregulated genes enriched nuclear division, organelle fission, chromosomal segregation, mitotic nuclear division, and meiotic cell cycle process, in addition to cell cycle and nicotine addiction pathways (Figures 2E,F).

### Construction of the CIN-Related Dysregulated CeRNA Network

**FIGURE 3:**
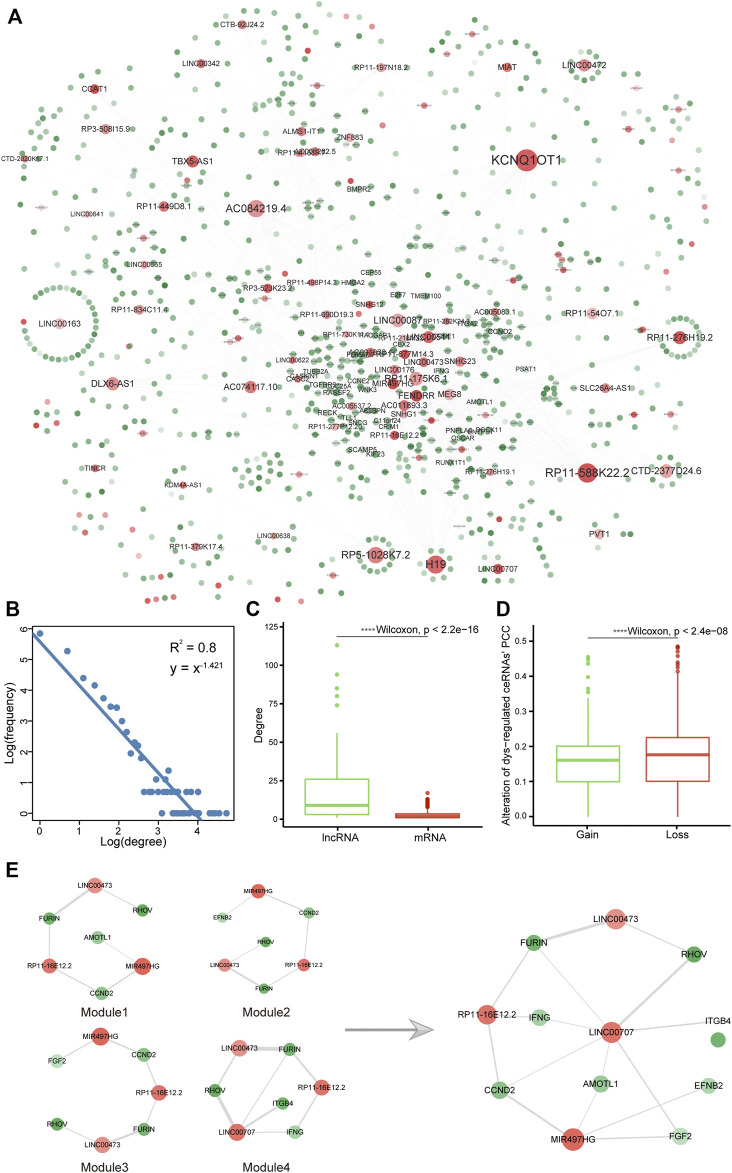
Construction of a CIN-related dysregulated ceRNA network. **(A)** CIN-related dysregulated double-weighted ceRNA network, in which mRNAs and lncRNAs were colored green and red, respectively. Node degree was indicated by the node size, and node weight and edge weight were indicated by node transparency and edge width, respectively. **(B)** Degree distribution of the network. Boxplots of **(C)** mRNA and lncRNA degree and **(D)** alteration of ceRNA dysregulation extent in CIN-High compared with CIN-Low samples. **(E)** Subnetwork CRDC connected by dense modules after screening and integration.

Due to the complex indirect regulation of lncRNAs for target mRNAs, we constructed a CIN-related dysregulated ceRNA network. The mRNAs and lncRNAs markedly changed on the expression level between LUAD versus adjacent normal samples were considered LUAD-associated genes and used to identify ceRNA pairs in CIN-Low and CIN-High samples, respectively. The ceRNA network contained 2,195 edges and 908 nodes composed of 787 mRNAs and 121 lncRNAs. We further constructed the double-weighted CIN-related dysregulated ceRNA network, in which the edge weight and node weight indicated the extent of dysregulation of ceRNA in CIN-High samples contrast with CIN-Low and the quantized value for RNA’s prognostic role in DSS, respectively (Figure 3A, Supplementary Table S2, details were prescribed in Materials and Methods). The network had a typical biological network property, a scale-free characteristic, and followed power-law distribution with R^2^ being 0.80 (Figure 3B). Network topological analysis suggested that lncRNAs may play an important role in regulating mRNA expression indirectly by the ceRNA crosstalk mechanism to exert biological functions in spite of low coding potential (Figure 3C and Supplementary Figures S2A–C).

In this work, the dysregulation type of ceRNA was defined as gain (PCC in the CIN-High group were higher than that in CIN-Low) or loss (PCC in CIN-High patients was smaller than that in CIN-Low). Among 2,195 ceRNAs, 828 of them were type of gain; however, the majority were loss (accounted for 62.3%), which suggested that most of ceRNAs no longer maintained previous correlation in the CIN-High group. PCC of gain pairs raised from 0.071 ± 0.115 to 0.224 ± 0.078 and in loss pairs reduced from 0.262 ± 0.097 to 0.090 ± 0.124. The extent of alteration in loss pairs had a greater alteration extent than that in the gain pairs (Supplementary Figures S2D,E and Figure 3D). BP functions were enriched by Metascape, in which gain and loss ceRNAs showed relevance to mitotic cell cycle, tube morphogenesis, regulation of cell cycle process, negative regulation of cell differentiation, epithelial cell differentiation, regulation of kinase activity, and cell adhesion, especially immune system development (Supplementary Figure S2F). It is implied that CIN-related dysregulated ceRNAs were involved with functions related to tumorigenesis and immunological functions.

### Filtration for Double-Weighted Dense Modules

We wish to seek a collection of ceRNAs possessing a high prognostic value and a great level of variation in samples with high and low CIN; therefore, we adopted the method of dmGWAS_3.0. The method balanced the weight of nodes and edges for scoring the modules and implemented a greedy algorithm to search for dense modules. The parameters were set as follows: *λ* for balancing weight of nodes and edges was set to “default” calculated as 0.44; the new nodes search range shortest path *d* = 1; *r* was decided as 0.1 which decides the magnitude of increment. A total of 651 modules were obtained by this method. These modules covered 776 nodes in the network, which demonstrated that these modules had the characteristic of high repeat coverage. Therefore, we screened and integrated these modules. First, 65 modules contained 83 non-repetitive RNAs with the top 10% highest module scores were selected, which displayed significant overlap with CIN70 genes on account of having four common mRNAs (HDGF, OIP5, CDK1 and CEP55, hypergeometric enrichment test *p* = 1.86e-05). Second, a random perturbation strategy was used to screen out the non-randomized prognostic modules, and only four modules with random *p* < 0.01 were left. It has been observed that they shared common RNAs, indicating that these modules can be linked to be a subnet in the dysregulated network. Therewith, the subnet consisting of these modules was extracted containing eight mRNAs (AMOTL1, EFNB2, FGF2, FURIN, CCND2, IFNG, ITGB4, and RHOV) and 4 lncRNAs (LINC00473, LINC00707, MIR497HG, and RP11-16E12.2) (Figure 3E). We further named the subnet as CRDC (CIN-related dysregulated ceRNAs).

### Formulation and Evaluation of the CRDC Risk Score and Prognostic Analysis

**FIGURE 4:**
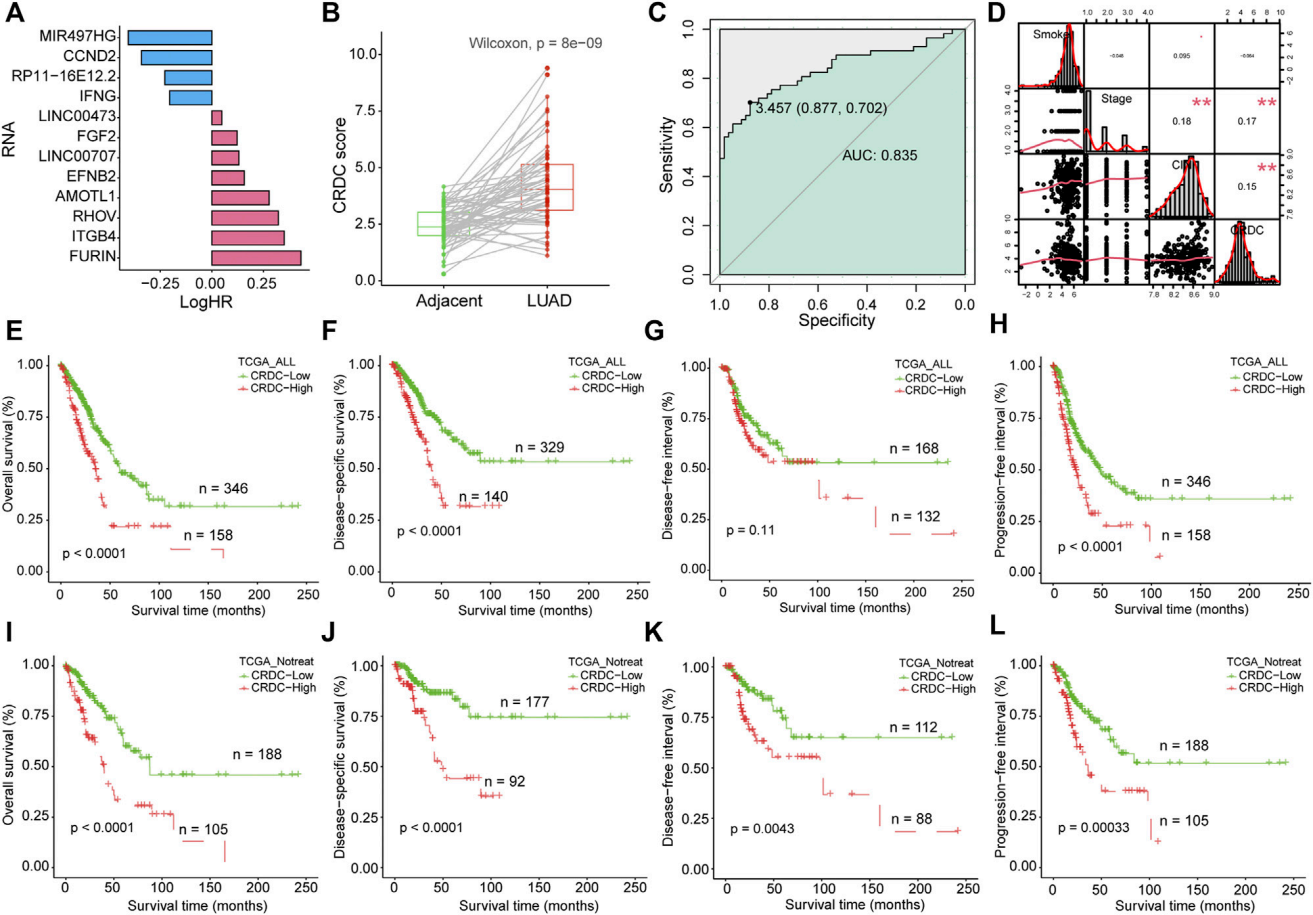
Formulation of the CRDC risk score and prognostic analysis. **(A)** Barplot depicted the log-transformed hazard ratio of CRDC genes in the univariate Cox regression model. **(B)** Boxplot for CRDC of LUAD and adjacent paired samples. The green column indicated adjacent samples, and the red column indicated LUAD tissues. **(C)** ROC curve of CRDC in discriminating LUAD patients from adjacent control samples. The green color indicated AUC. **(D)** Spearman correlation analysis of smoking, clinical stage, CIN, and CRDC risk score. Kaplan–Meier survival curves of the CRDC risk score in **(E–H)** all LUAD patients (TCGA_ALL, for short) and **(I–L)** patients without postoperative treatment (TCGA_Notreat, for short).

The CRDC risk score for each sample was calculated as cumulative sum of expression values of 12 RNAs multiplied by log-transformed hazard ratios from univariate Cox regression analysis in non-postoperative treatment patients (the univariate Cox regression results of RNAs are shown in Figure 4A and Supplementary Table S3). Most of the CRDC genes tended to be risk factors of LUAD prognosis, except for MIR497HG, CCND2, RP11-16E12.2, and IFNG. Studies have shown that CRDC genes were certified related with tumor suppression or progression which is consistent with our research (Supplementary Table S4). In addition, the protein expression levels of partial CRDC genes were further explored by representative immunohistochemistry (IHC) images from the Human Protein Atlas (HPA) database (Sjostedt et al., 2020). In total, five CRDC proteins, FURIN, ITGB4, RHOV, EFNB2, and AMOTL1, were not detected in normal lung tissues, however, expressed in lung cancer samples. Also, IFNG had a reverse situation (Supplementary Figure S3).

The CRDC risk scores in LUAD were significantly higher than adjacent normal in all (Wilcoxon signed-rank test, *p* = 1.1e-15, Supplementary Figure S4A) and paired samples (Wilcoxon signed-rank test, *p* = 8e-9, Figure 4B), and it can distinguish the two types of samples clearly (AUC = 0.835, Figure 4C). The higher CRDC score related to the worse clinical stage (Kruskal–Wallis test, *p* = 0.0012, Supplementary Figure S4B). Spearman rank correlation analysis was performed on the CRDC risk score, CIN score, clinical stage, and smoking exposure in pack-years. There was no significant correlation between smoking and CRDC risk score or clinical stage (*p* > 0.05), but a margin significant positive correlation with CIN risk scores, which was consistent with the findings that smoking was probably associated with chromosomal instability (Saletta et al., 2007; Li E. et al., 2014). What was noteworthy is that the CIN score, clinical stage, and CRDC risk score were significantly positively correlated, which indicated that the higher the CRDC risk score, the higher the chromosomal instability and the worse clinical stage (Figure 4D). Also, the high CRDC score was associated with poor OS, DSS, and PFI in all LUAD patients (Figures 4E–H). Because postoperative treatment may have some effect on clinical outcomes which was taken into account in the assessment of the impact of CRDC on prognosis. Among samples without postoperative treatment, the OS, DSS, DFI, and PFI of the CRDC high risk group were significantly worse than those of the low risk group (Figures 4I–L). These results have shown that LUAD maintained higher CRDC scores than adjacent normal tissues, such as CIN. Also, the higher CRDC score was concordance with higher CIN and worse clinical stage. The impact of CRDC on prognosis was rarely affected by the postoperative treatment in the TCGA LUAD cohort.

### Prognostic Independence Evaluation and External Prognosis Validation of CRDC

**FIGURE 5:**
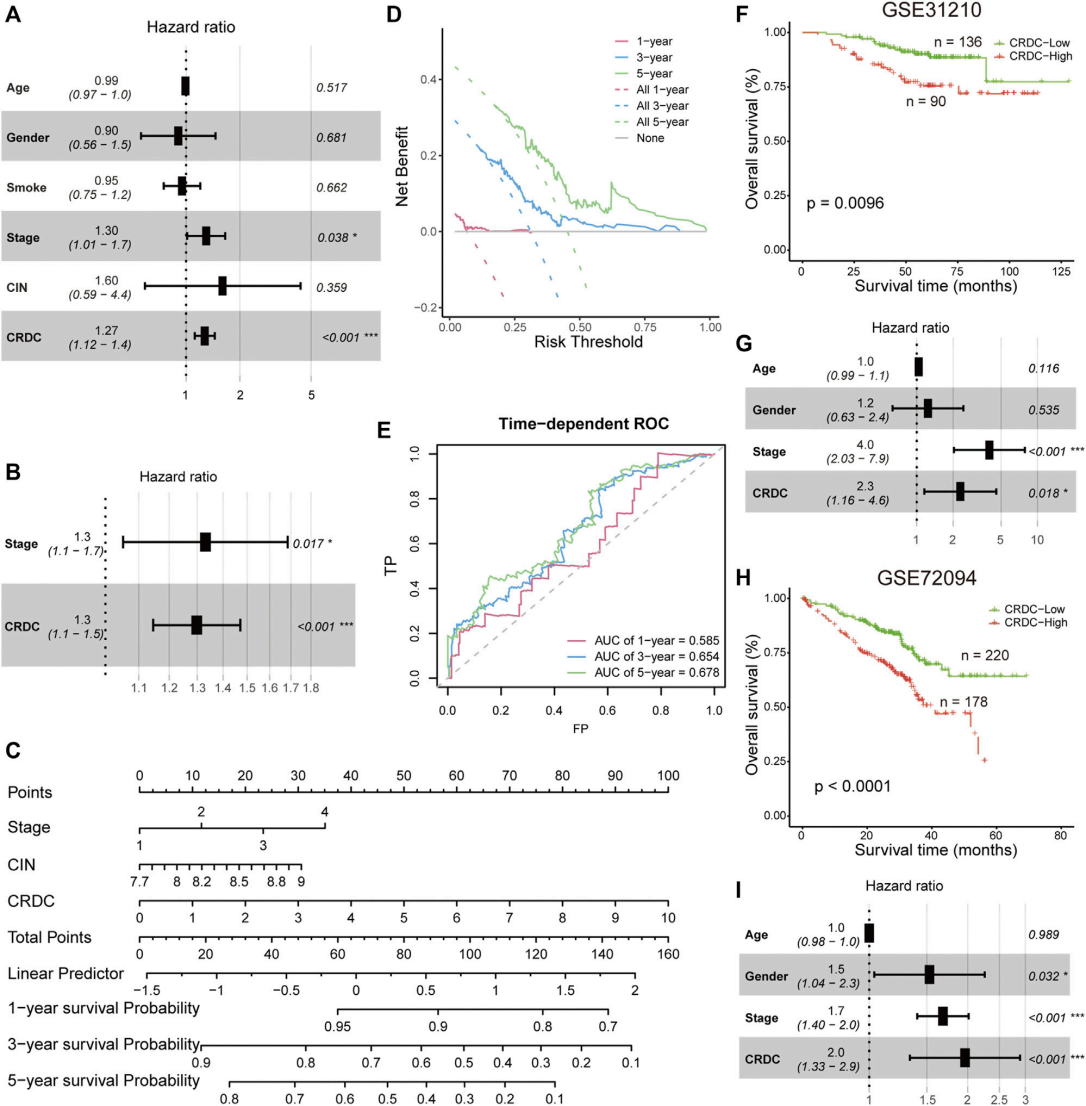
Prognostic independence evaluation and external validation. **(A)** Forest plot for the multiple Cox proportional hazards model in TCGA patients. **(B)** Forest plot for the multiple Cox proportional hazards model contained the clinical stage and CRDC risk score in TCGA patients **(C)** Nomogram constructed by the clinical stage and CRDC risk score for predicting 1, 3, and 5 years DSS. **(D)** Decision curve of the nomogram for 1, 3, and 5 years DSS. **(E)** Time-dependent ROC of the CRDC risk score for predicting 1, 3, and 5 years DSS outcome. Kaplan–Meier survival curves of CRDC-High and CRDC-Low patients and forest plots for the multiple Cox proportional hazards model in **(F,G)** GSE31210 and **(H,I)** GSE72094 datasets.

In this part, prognostic independence and robustness of the CRDC score will be tested. In the TCGA LUAD cohort, multivariate Cox proportional hazard analysis indicated that CRDC was an independent risk factor of DSS considered with factors of age, gender, smoking, clinical stage, and CIN score (*p* < 0.001, HR = 1.27, 95%CI: 1.12–1.4, Figure 5A). On account of the multicollinearity between variables (Figure 4D), we utilized stepwise multivariate Cox hazard regression to overcome this issue. The result showed that age, gender, smoking, and CIN had no significant effect on DSS. Only the clinical stage (*p* = 0.017, HR = 1.3, 95%CI: 1.1–1.7) and CRDC risk score (*p* < 0.001, HR = 1.3, 95%CI: 1.1–1.5) tended to be the independent risk factors for DSS, with the CRDC being the most significant factor (Figure 5B). A 1, 3, and 5 years DSS nomogram was constructed by the clinical stage and CRDC risk score showed that CRDC remained certain reference and prediction value for clinicians (corrected C-index = 0.661, Figure 5C). The CRDC score had a great benefit for predicting DSS outcome, which was observed from the decision curve analysis (DCA) of the nomogram (Figure 5D). Time-dependent AUCs of the CRDC score in predicting 1, 3, and 5 years DSS were, respectively, 0.585, 0.654, and 0.678 (Figure 5E). Moreover, in two external GEO LUAD datasets (GSE31210 and GSE72094), the CRDC score still performed a stable and robust prognostic value in predicting patients’ overall survival and acted as an independent risk factor for prognosis (Figures 5F–I). In short, we demonstrated the robust performance of CRDC for LUAD outcome prediction in TCGA and two GEO cohorts.

### Exploration of CRDC-Related Biological Functions Indicating the Immunology Correlation

**FIGURE 6:**
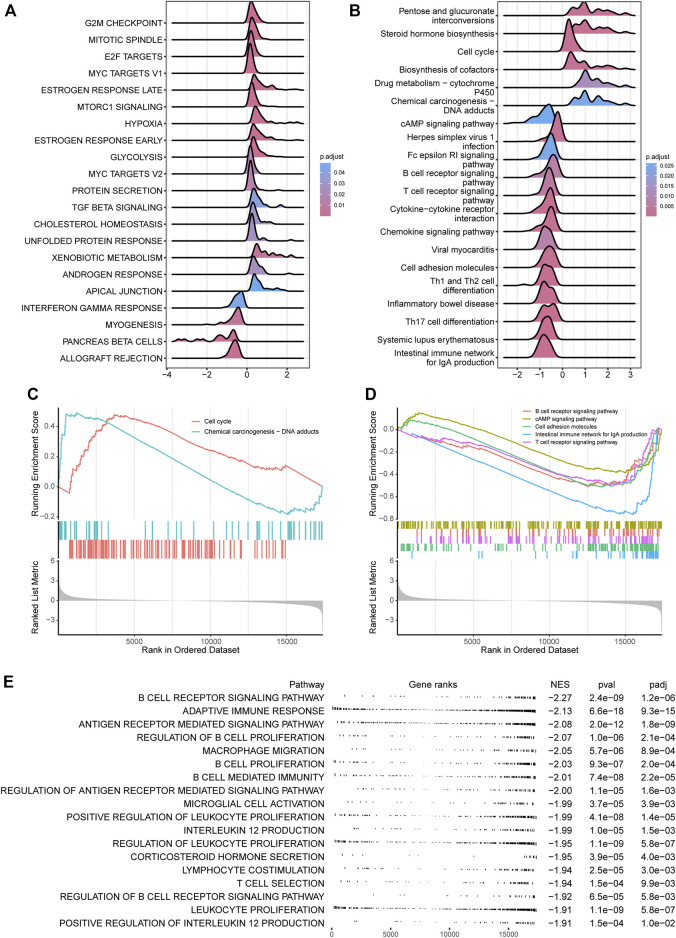
CRDC relevant functional analysis. Ridgeline plots for log2FC (CRDC-High versus CRDC-Low) distribution of core-enriched genes in **(A)** hallmarks and **(B)** KEGG pathways based on the GSEA method. GSEA plots for **(C)** positive and **(D)** negative KEGG pathways associated with CRDC. **(E)** Table of the CRDC-related negative GO (BP) enrichment graph.

For investigating CRDC-related functions, differential gene analysis between CRDC-High and CRDC-Low patients was performed by DESeq2 at first. We identified that the CRDC had positive connections with multiple hallmarks such as G2M checkpoint, E2F targets, and MYC targets. Targeting WEE1, which was crucial in the G2M cell-cycle checkpoint arrest for DNA repair before mitotic entry, for inhibition and compromising the G2M checkpoint presents an opportunity to potentiate therapy (Matheson et al., 2016). Alterations in one or more key components of the core transcriptional machinery formed by the cyclin-dependent kinase (CDK)-RB-E2F axis result in heightened oncogenic E2F activity, leading to uncontrolled proliferation in cancer (Kent and Leone, 2019). Genetic deregulation of MYC expression and loss of checkpoint components, such as TP53, permit MYC to drive malignant transformation in cancer (Stine et al., 2015). On the contrary, Interferon gamma is a member of the type II interferon class, secreted by cells of both the innate and adaptive immune systems, and is crucial in antitumor response (Shankaran et al., 2001). The downregulation of interferon gamma response activity indicated that there was some connection between CRDC and immunity (Figure 6A). Also, we found high activity in the cell cycle and chemical carcinogenesis pathways, conversely, low activity in the cAMP signaling pathway, cell adhesion molecules, intestinal immune network for IgA production, B cell receptor signaling pathway, and T cell receptor signaling KEGG items (Figures 6B–D). CAMP signaling increases histone deacetylase 8 (HDAC8s) expression through the Epac–Rap1–Akt pathway leading to augmenting cisplatin-induced apoptosis (Park and Juhnn, 2017) and inhibits radiation-induced ATM phosphorylation promoting apoptosis in lung cancer (Cho et al., 2014). Some cell adhesion molecules are now considered clinical biomarkers in multiple tumor types, contributing to carcinoma progression and metastasis (Beauchemin and Arabzadeh, 2013; Smart et al., 2021). Researchers have found that the intestinal immune network for IgA production was significantly dysregulated in lung metastases from colorectal cancer (Shen et al., 2021). Decreasing activity of B cell and T cell receptor signaling implied that high CRDC risk may be related to reduced humoral immunity and cell-mediated immunity. We observed that CRDC displayed relevance to plenty of immunologic signatures (Supplementary Figures S4D,E); likewise, the suppression of immunity function appeared in the BP GSEA results (Figure 6E), which provided further evidence that CRDC was related to immunologic function. These function enrichment analyses showed that genes divided by the CRDC risk score enriched a number of known carcinoma-associated biological functions and reminded us of the immunological correlation with CRDC at the same time.

### Immune Infiltration Microenvironment Characteristics Related With CRDC

**FIGURE 7:**
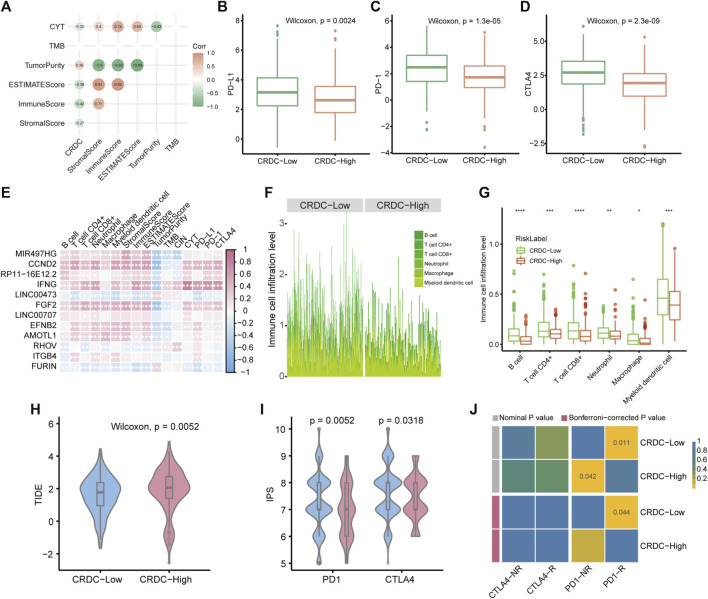
CRDC-related immune infiltration characteristics. **(A)** Spearman correlation analysis plot of the correlativity of the CRDC score, StromalScore, ImmuneScore, ESTIMATEScore, TumorPurity, TMB, and CYT. Boxplots described the expression difference of **(B)** PD-L1, **(C)** PD-1, and **(D)** CTLA4 between CRDC-High and CRDC-Low samples. **(E)** Spearman correlation heatmap of CRDC genes and immune microenvironment cell infiltration, StromalScore, ImmuneScore, ESTIMATEScore, TumorPurity, TMB, CIN, CYT, PD-L1, PD-1, and CTLA4. **(F,G)** Immune microenvironment cell infiltration level in CRDC-High and CRDC-Low samples. The violin plots displayed the TIDE **(H)** and IPS **(I)** scores between CRDC-High and -Low groups. **(J)** Heatmap of comparison and identification of common subtypes between groups divided by CRDC risk and response for ICB therapy. NR and R represented not respond and respond to immunotherapy, respectively.

The functional analysis mentioned previously indicated that CRDC may correlate with immunological function changes; therefore, we implemented further exploration of immune characteristics in LUAD samples. CRDC correlated with tumor purity positively and had negative correlation with CYT, ESTIMATEScore, ImmuneScore, and StromalScore (Spearman correlation test, *p* < 0.05, Figure 7A). LUAD patients with lower CRDC scores exhibited higher PD-L1, PD-1, and CTLA4 expression levels than CRDC-High patients (Wilcoxon signed-rank test, *p* < 0.05, Figures 7B–D). The correlation heatmap showed that multiple CRDC genes have a significant correlation with immune cell proportion in the microenvironment (Figure 7E). Also, patients with lower CRDC scores had more B cell, T cell CD4^+^, T cell CD8^+^, neutrophil, macrophage, and myeloid dendritic cell infiltration than patients with higher CRDC scores (Figures 7F,G). The TIDE and IPS scores exhibited differences in patients distinguished by the CRDC score, which displayed that LUAD patients with decreased CRDC risk scores had lower immune dysfunction and exclusion score as well as more possibility to benefit from immune checkpoint therapy (Figures 7H,I). In addition, based on the common subtype comparison analysis between two CRDC groups and melanoma samples with the information of immunotherapy reaction (Roh et al., 2017; Chen et al., 2021), we could infer that patients with low CRDC risk were more likely to respond to anti-PD1 therapy (Bonferroni-corrected *p* = 0.044), yet high CRDC group may be resistant to this form of treatment (Nominal *p* = 0.042, Figure 7J). In brief, we revealed that CRDC had relevance to immune characteristics, and patients with low CRDC risk exhibited greater immunocompetence than those with high risk.

## Discussion

Previous studies have shown that CIN was closely related to tumorigenesis. Gain of chromosomes showed increased DNA damage and sensitivity to replication stress, thereby promoting genomic instability and possibly contributing to tumorigenesis (Passerini et al., 2016). Based on discussion of mathematical models of situations in which inactivation of one or two tumor suppressor genes is required for tumorigenesis; CIN will arise before inactivation of the first suppressor genes, therefore initiating the mutational sequence that leads to cancer (Michor et al., 2005). In restricted genetic and cellular contexts, the mitotic checkpoint protein insufficiency can cause whole chromosome instability and drive tumor formation through tumor suppressor gene loss of heterozygosity (Baker et al., 2009). In this work, we investigated the CIN characteristics in 513 TCGA LUAD patients and found that the extent of CIN in LUAD was significantly higher than that in adjacent normal samples, and it can be used to strictly separate the tumor from adjacent normal samples (Figures 2A,B). CIN 70 marker genes expressed a high level in LUAD (Figure 2D). Also, CIN was related to high clinical stage as well as poor survival in LUAD patients (Figure 2C and Supplementary Figure S1). Functional analysis results indicated that CIN may have an effect on tumor progression by promoting nuclear division, organelle fission, chromosomal segregation, mitotic nuclear division, and meiotic cell cycle process, in addition to cell cycle and nicotine addiction pathways (Figures 2E,F). These results were identical with the previous theory that tumors are generally characterized by genomic instability.

For the past few years, the molecular regulatory mechanism of ceRNA has been proved to play a crucial role in development of diseases, especially cancers and their dysregulation may conduce to tumor pathogenesis. In our previous work, we have constructed a clear cell kidney carcinoma dysregulated ceRNA–ceRNA network and identified two dysregulated patterns of ceRNAs interaction (gain and loss), which were demonstrated to be able to distinguish normal samples from cancer samples (Wang et al., 2018). The ceRNA dysregulation mechanism in LUAD needed expansion, and no research has explored characteristics of ceRNA alteration in the CIN level yet. In the present work, we explored CIN-related dysregulated ceRNAs in LUAD aiming for acquiring a prognosis indicator for the first time. Based on differential expression genes (mRNAs and lncRNAs) in LUAD compared to adjacent samples, we constructed a double-weighted CIN-related dysregulated ceRNA network. The edge weight in the network was the alteration of ceRNA PCC in CIN-High compared with CIN-Low patients representing the extent of ceRNA dysregulation. Also, the node weight represented the potency of the gene in predicting LUAD patient’s outcome. At this step, we adopted the samples without postoperative treatment eliminating the possible impact of treatment on prognosis. Also, we used DSS as a measure of prognosis in consideration of its definition, death from the diagnosed cancer type, has much greater relevance with cancer-associated clinical outcome than OS in which the endpoint may record non-cancer causing death (Liu et al., 2018). Based on the dysregulated network we built, 62.3% of the ceRNAs maintained a weaker co-expression relationship in the CIN-High than CIN-Low group (Figure 3D and Supplementary Figures S2D,E). Also, the greedy search algorithm was applied to detect modules with high double weight, whereafter we obtained a subnetwork CRDC formed by 12 genes after module screening and integrating (Figure 3E).

The CRDC risk score was higher in LUAD samples than that in adjacent normal samples and could separate the two types of samples, which was resembled with CIN (Figure 4B,C). Subsequently, we laid special stress on testing and evaluating the prognostic prediction efficiency of CRDC. The results implied that the high CRDC score can serve as an indicator for poor OSS, DSS, and PFI in patients with or without postoperative treatment and a key independent risk factor in TCGA and two GEO datasets (Figure 4E–L, Figure 5). In the multivariate Cox proportional hazard model, CIN did not show as an independent indicator for predicting DSS. It demonstrated that prognostic efficacy of CIN was not as stable as that of CRDC. We suspected that there was probably a connection with the dual natures and complicacy of CIN in cancer progressing. Extreme CIN was related to long-term survival in primary breast cancer (Roylance et al., 2011). In addition, the paradoxical and nonmonotonic relationship between CIN and prognosis was observed in ovarian, gastric, and non-small-cell lung tumors (Birkbak et al., 2011). CIN plays a multifaceted role in cancer, and its microenvironment, for instance, by introducing double-stranded DNA into the cytosol, CIN could engage the cGAS–STING antiviral pathway to facilitate inflammatory signaling (Bakhoum and Cantley, 2018).

The functional analysis results indicated that CRDC was probably relevant with biological function terms associated with tumor progression. For instance, high activity in G2M checkpoint, E2F targets, MYC targets, cell cycle, and chemical carcinogenesis, and low activity in the cAMP signaling pathway and cell adhesion molecules in opposite. At the same time, a number of immune-associated terms, such as interferon gamma response and B cell and T cell receptor signaling, exhibited a negative changing trend. These results suggested that the poor prognosis caused by CRDC may be achieved by promoting cell proliferation and migration as well as reducing antitumor immune response.

Based on the possibility of the connection between CRDC and immunity from the functional analysis, we consulted literatures and found that several CRDC genes have been proven to be associated with immunologic function. LINC00473 silencing enhanced miR-195-5p-targeted downregulation of PD-L1 in pancreatic cancer may block the cancer progression (Zhou W. Y. et al., 2019). The proprotein convertase furin was negatively correlated with immune cell infiltration in triple negative breast cancer fitting with our correlation analysis about the CRDC genes and immune features (Figure 7E). Also, furin deficiency in T cells decreased Tregs resulting in CD8^+^ T cell activation and IFN-γ upregulation (He et al., 2020). IFN-γ can augment immune function, however, induce expression of PD-L1 by which IFN-γ impairs antitumor immunity (Mandai et al., 2016). Also, tumor IFNG signaling blocking improves ICB response by CD8^+^ T cell and NK/ILC1-mediated killing (Benci et al., 2019). We found a higher expression level of IFNG and immune checkpoint genes in the CIN-Low group versus CIN-High group (Supplementary Figure S4C and Figures 7B–D), which suggested that CIN-Low patients had possibility to benefit from ICB. Also, Patients with low CRDC risk had lower immune dysfunction and exclusion score as well as higher CYT, immunophenoscore, and immune cell infiltration, which illustrated that these patients represented characteristics of “immune-hot” (Figures 7A,F–I). Immunotherapy response prediction offered evidence of the anti-PD1 therapy potential applicability for CIN-Low patients (Figure 7J).

There are two limitations in our work. We adopted the patients in TCGA cohort without postoperative treatment to evaluate prognosis for excluding the impact of treatment as much as possible. However, these untreated samples may be derived from information records missing, and we could not acquire the unambiguous treatment information from the external GEO verification datasets. In addition, the CRDC-related biological functions and immunological characteristics needed in-depth experimental verification.

## Conclusion

In conclusion, we analyzed the CIN feature and constructed a double-weighted CIN-related dysregulated ceRNA network. Based on this network, a potential marker CRDC composed of 12 RNAs was acquired which could distinguish LUAD samples from adjacent normal tissues and correlated with multiple tumorigenic hallmarks and pathways. Also, CRDC probably serves as an indicator for evaluating prognosis and recommending immune checkpoint inhibitor therapy. This study will offer a novel perspective for understanding the molecular action in LUAD tumorigenesis and progression.

## Data Availability

The original contributions presented in the study are included in the article/Supplementary Material, further inquiries can be directed to the corresponding authors.
