# Supplementary material for: Prognostic Implications and Immune Infiltration Characteristics of Chromosomal Instability-Related Dysregulated CeRNA in Lung Adenocarcinoma
Source: Front Mol Biosci. 2022 Mar 28;9:843640. doi: 10.3389/fmolb.2022.843640 (PMC8995899; doi:10.3389/fmolb.2022.843640)
Supplement: Supplementary file 1 [file DataSheet1.PDF]

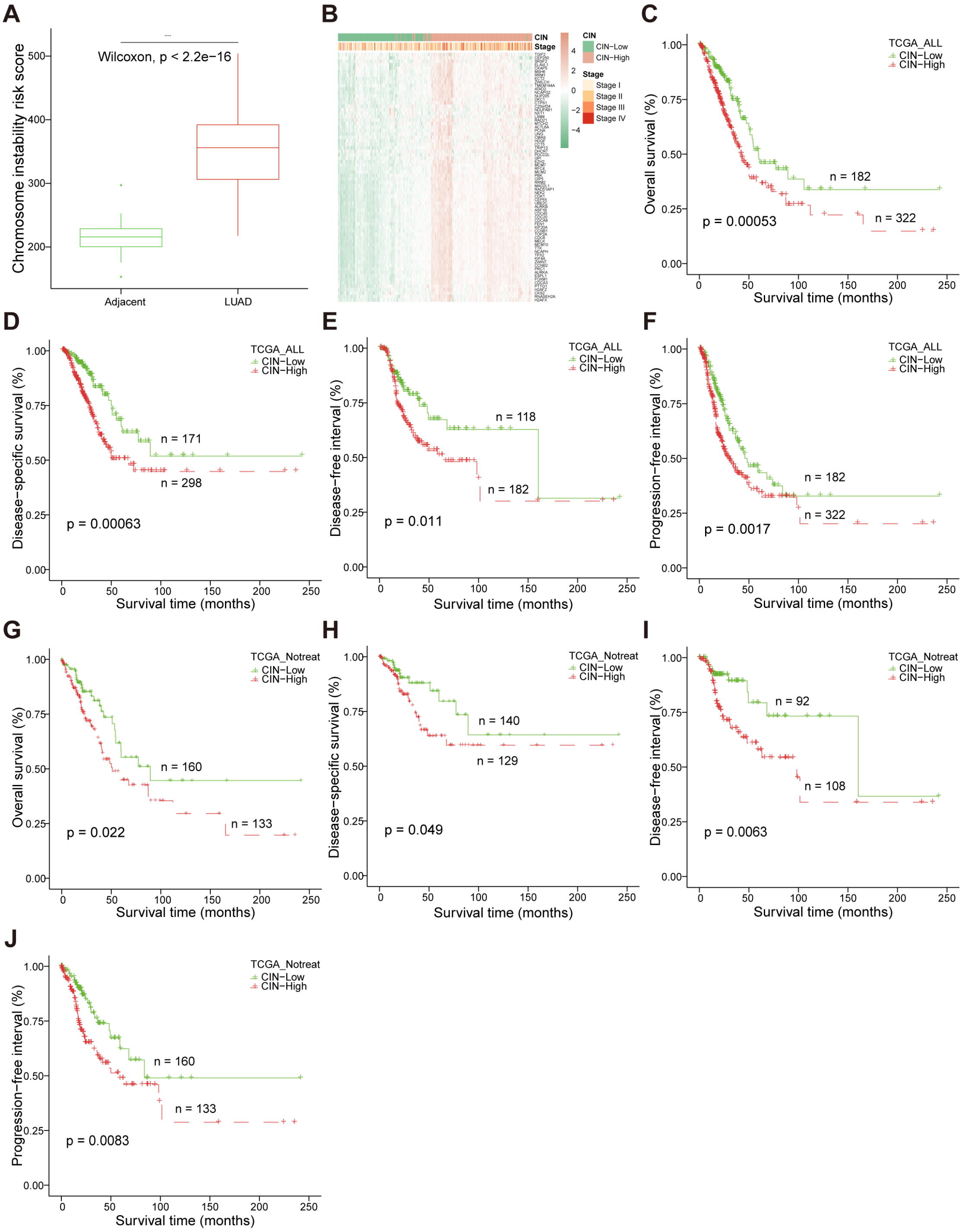

Figure S1. Characteristics and survival analysis of chromosomal instability in lung adenocarcinoma. (A) Boxplot for CIN scores of LUAD and adjacent samples. The green column indicated adjacent samples, and the red column indicated LUAD tissues. (B) Heatmap of CIN70 genes expression level in LUAD samples. Kaplan–Meier survival curves of the CIN risk score in (C) OS, (D) DSS, (E) DFI, and (F) PFI of all patients (TCGA_ALL, for short). Kaplan–Meier survival curves of CIN risk score in (G) OS, (H) DSS, (I) DFI and (J) PFI in patients without postoperative treatment (TCGA_Notreat, for short).

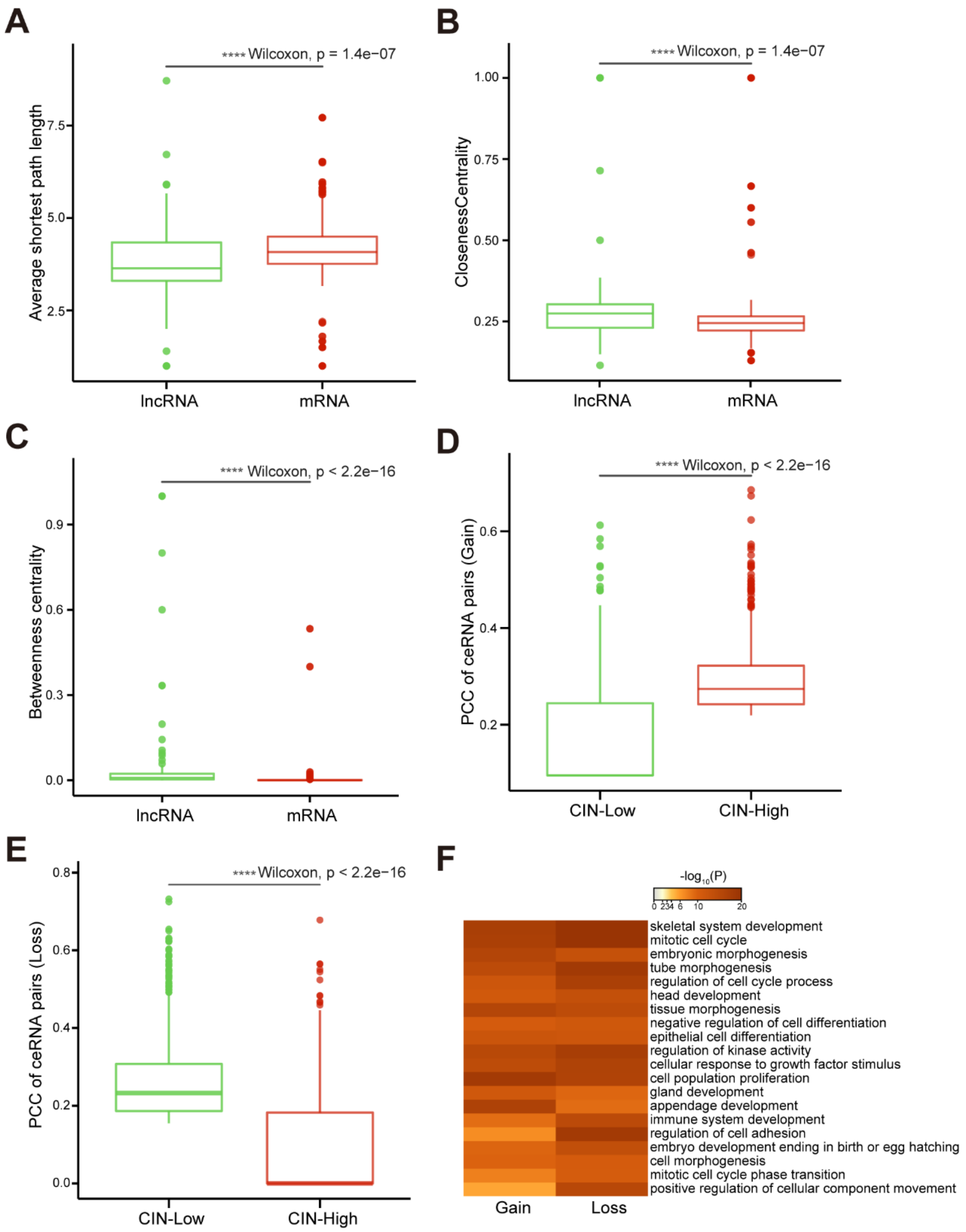

Figure S2. Characteristics of CIN-related dysregulated ceRNA network. (A-C) Network topological analysis for lncRNAs and mRNAs in CIN-related dysregulated ceRNA network. (D-E) Boxplots depicted alteration of dysregulation of ceRNAs in CIN-High and CIN-Low samples. (F) Heatmap of BP function enrichment results of gain and loss ceRNAs implemented by Metascape.

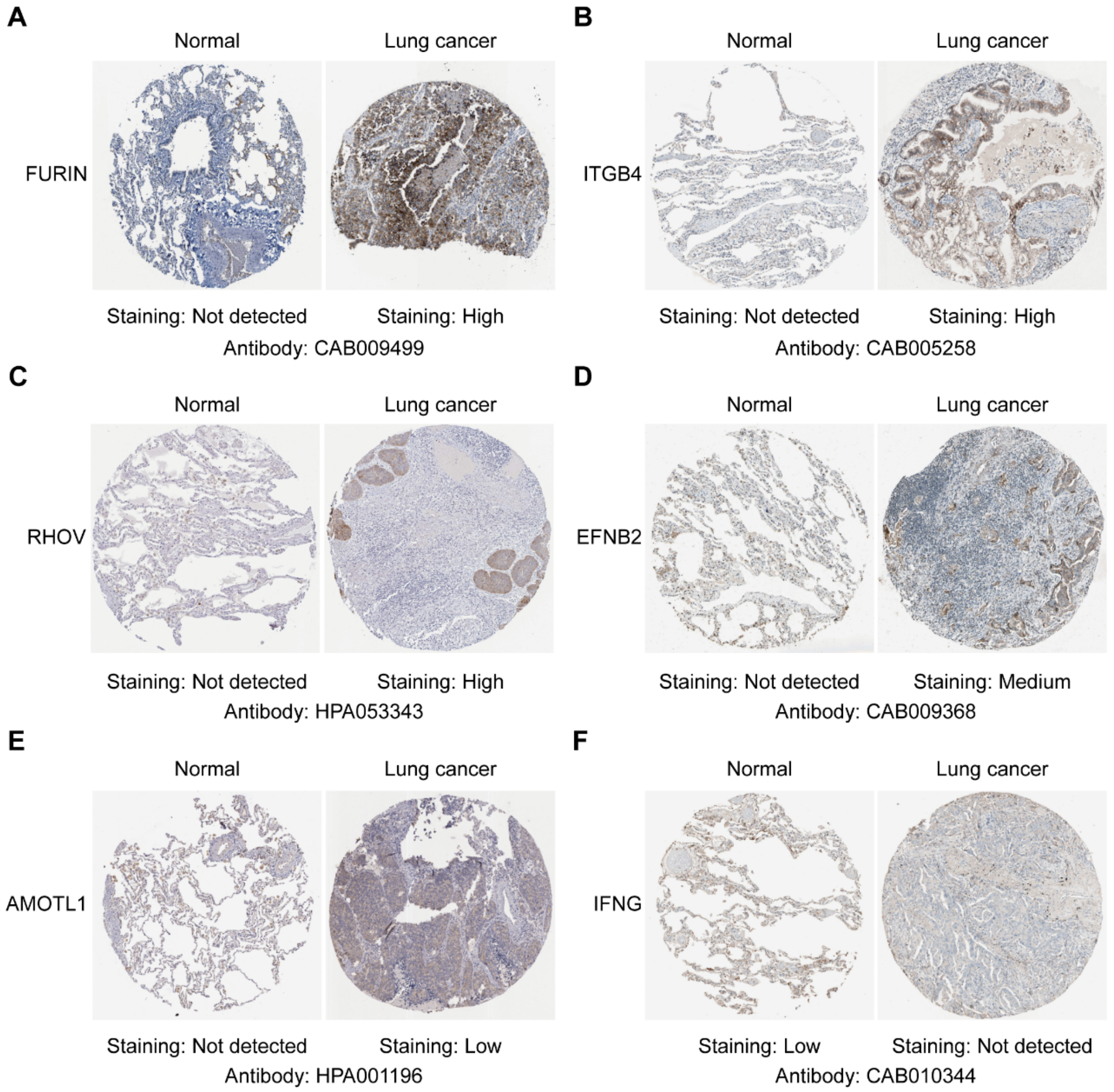

Figure S3. IHC analysis of proteins in CRDC genes. IHC results of six differentially expressed proteins in lung cancer versus normal samples from the Human Protein Atlas database.

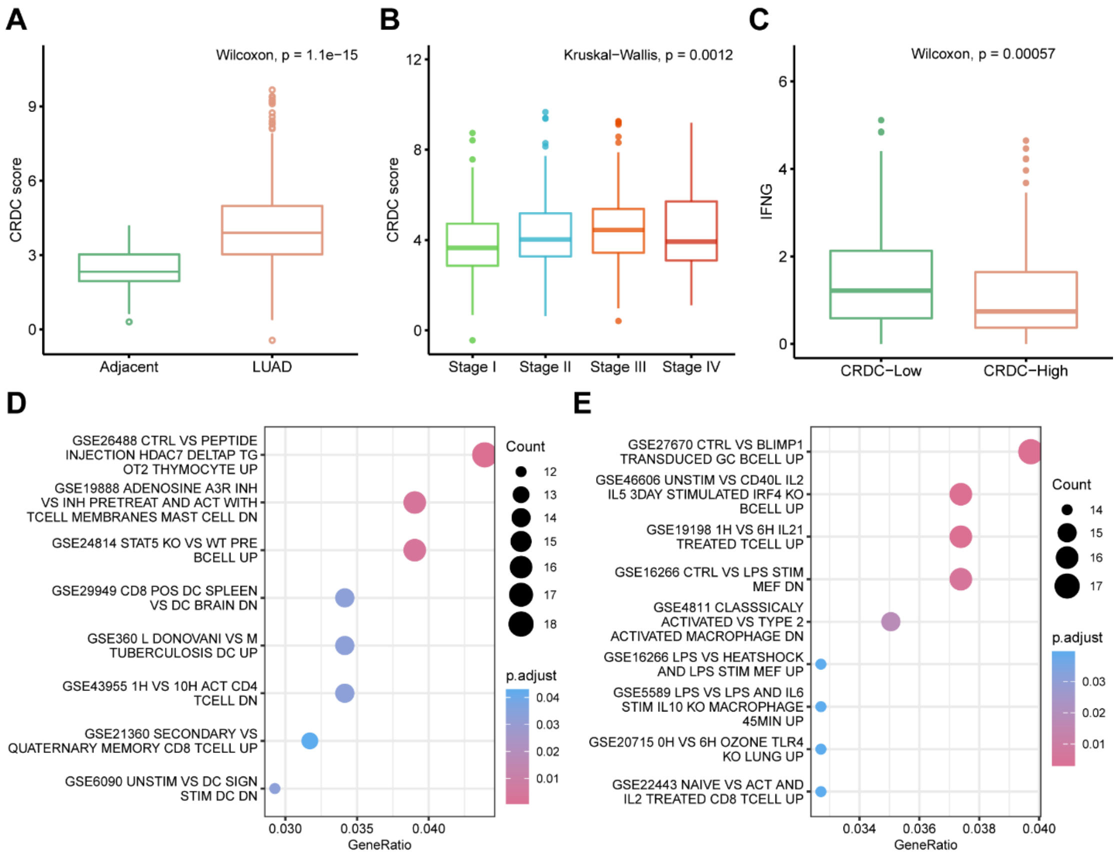

Figure S4. CRDC score features and immunologic signature gene sets enrichment analysis. (A) Boxplots for CRDC scores in LUAD versus adjacent normal samples and (B) different clinical stages. (C) Boxplot described the expression difference of IFNG between CRDC-High and CRDC-Low samples. Dot plot of (D) up-regulated and (E) down-regulated genes in CRDC-High versus CRDC-Low group enrichment results for immunologic signature gene sets.

Table S1. Sample size of two GEO datasets

Data Set	Platforms	Samples with survival information	Number of genes overlap with CRDC
GSE31210	GPL570	226	9
GSE72094	GPL15048	398	9

Table S3. Results of CRDC genes in univariate Cox regression model

RNA name	RNA type	HR	95%CI	p-value	LogHR
MIR497HG	lncRNA	0.667	0.482-0.922	**0.0143**	-0.405
CCND2	mRNA	0.711	0.524-0.965	**0.0284**	-0.341
RP11-16E12.2	lncRNA	0.796	0.643-0.985	**0.0358**	-0.228
IFNG	mRNA	0.815	0.598-1.11	0.195	-0.205
LINC00473	lncRNA	1.05	0.976-1.14	0.183	0.0488
FGF2	mRNA	1.13	0.788-1.61	0.513	0.122
LINC00707	lncRNA	1.14	1.01-1.29	**0.0341**	0.131
EFNB2	mRNA	1.17	0.889-1.53	0.265	0.157
AMOTL1	mRNA	1.32	0.975-1.78	0.0723	0.278
RHOV	mRNA	1.38	1.17-1.63	**0.000172**	0.322
ITGB4	mRNA	1.42	1.15-1.77	**0.00128**	0.351
FURIN	mRNA	1.54	1.18-2	**0.00143**	0.432

Table S4. Experimental validation literatures for the role of CRDC genes in cancers

RNA name	RNA type	PMID	Summary
MIR497HG	lncRNA	33363213; 34664678	Silencing of lncRNA MIR497HG promotes bladder cancer cell growth, migration, and invasion in vitro through promoting the crosstalk between Hippo/Yap and TGF-β/Smad signaling; MIR497HG served as a tumor suppressor and miR-497/MIR497HG inhibits glioma cell proliferation by targeting CCNE1 and the miR-588/TUSC1 axis.
CCND2	mRNA	30308939	CCND2 expression inhibited lung and breast cancer cell growth and migration ability, and hypermethylation of CCND2 in lung and breast cancer is a potential biomarker and drug target.
IFNG	mRNA	29516506	IFN-γ-mediated inhibition of lung cancer correlates with PD-L1 expression and is regulated by PI3K-AKT signaling.
LINC00473	lncRNA	34091587	Overexpression of LINC00473 restricts miR-502-3p upregulates KMT5A expression, and promotes the expression of cyclin D1 and CDK2, which is conducive to the cell cycle process in invasive pituitary adenoma.
FGF2	mRNA	29635378	FGF2 and EGF induce epithelial-mesenchymal transition in malignant pleural mesothelioma cells via a MAPKinase/MMP1 signal;
LINC00707	lncRNA	30502359	LINC00707 promotes proliferation and metastasis of gastric cancer by interacting with mRNA stabilizing protein HuR.
EFNB2	mRNA	30662604	EFNB2 knockdown inhibited cell proliferation, migration and invasion in pancreatic ductal adenocarcinoma.
AMOTL1	mRNA	32313226	AMOTL1 plays an oncogenic role in gastric carcinogenesis through interacting with YAP1 and promoting its nuclear accumulation.
RHOV	mRNA	34326698; 33767988	RHOV promotes lung adenocarcinoma cell growth and metastasis through the JNK/c-Jun pathway; Overexpression of RhoV promotes the progression and EGFR-TKI resistance of lung adenocarcinoma.
ITGB4	mRNA	31843981; 29059156	Integrin β4-Targeted cancer immunotherapies inhibit tumor growth and decrease metastasis; By inducing integrin β4/FAK/SOX2/HIF-1α signaling pathway

			in gastric cancer, ECM1 promotes cell metastasis and glucose metabolism.
FURIN	mRNA	27584082; 32389711	Targeting proprotein convertases in furin-rich lung cancer cells results in decreased in vitro and in vivo growth; Loss of the proprotein convertase Furin in T cells represses mammary tumorigenesis in oncogene-driven triple negative breast cancer.
